# Whole-Genome Sequence Analysis, Probiotic Potential, and Safety Assessment of the Marine Bacterium *Paraliobacillus zengyii* CGMCC1.16464

**DOI:** 10.3390/md23050202

**Published:** 2025-05-07

**Authors:** Qianjin Fan, Mengqi Jiao, Haoyue Huangfu, Lan Chen, Beijie Li, Zhijie Cao, Xuelian Luo, Jianguo Xu

**Affiliations:** 1 School of Medicine, Nankai University, Tianjin 300071, China; fanqianjin95@126.com (Q.F.); haoyue3333@163.com (H.H.); 2 National Key Laboratory of Intelligent Tracking and Forecasting for Infectious Diseases, National Institute for Communicable Disease Control and Prevention, Chinese Center for Disease Control and Prevention, Beijing 102206, China; jiaomengqizc@163.com (M.J.); caozhijie@icdc.cn (Z.C.); 3 Center of Reverse Microbial Etiology, School of Public Health, Shanxi Medical University, Taiyuan 030001, China; chenlan5475@163.com (L.C.); libeijie0211@163.com (B.L.)

**Keywords:** *Paraliobacillus zengyii* CGMCC1.16464, marine bacterium, in vitro and in vivo safety assessments, whole-genome sequence analysis

## Abstract

*Paraliobacillus zengyii* CGMCC1.16464 (*P. zengyii*) is a novel antiviral probiotic candidate strain. To ensure its safety as a potential probiotic, a safety evaluation was conducted in this study. The safety and functional potential of *P. zengyii* were systematically assessed through genomic bioinformatics analysis, in vitro experiments, and acute oral toxicity tests in mice. Genomic analysis revealed that *P. zengyii* is rich in genes related to carbohydrate and amino acid metabolisms and carries genes encoding antimicrobial and antiviral agents (such as ectoine, type III polyketide synthase, and lasso peptides). It also expresses gastrointestinal tolerance-related proteins (ClpC, GroEL, and ClpP). Its resistance to polymyxins is an inherent trait with no risk of plasmid-mediated transfer. In vitro experiments confirmed that *P. zengyii* is somewhat tolerant to bile salts and acidic environments and does not exhibit hemolytic or gelatinase activity. Importantly, an acute oral toxicity test in mice revealed that after intervention with high, medium, or low doses, no significant abnormalities in the body weight, organ index, or tissue morphology of the mice were observed. In conclusion, *P. zengyii* exhibited good safety and probiotic potential in terms of genomic safety, metabolic function, and in vitro and in vivo toxicities, providing a theoretical basis for the development of novel functional probiotics.

## 1. Introduction

The genus *Paraliobacillus* belongs to the family Bacillaceae of the phylum Firmicutes. Members of this genus are Gram-positive, rod-shaped, halotolerant, facultatively anaerobic, spore-forming marine bacteria. *Paraliobacillus ryukyuensis* sp. nov. was the first new species of this genus, initially isolated from decomposed marine algae from the Okinawa waters in Japan [1]. Studies have shown that this strain can produce a variety of beneficial short-chain fatty acids (SCFAs), including formate and acetate [1]. These metabolic products may have potential probiotic effects on gut health, such as enhancing the intestinal barrier, reducing inflammation, and inhibiting the growth of harmful microbiota [2,3]. *Paraliobacillus zengyii* CGMCC1.16464 (*P. zengyii*) is another member of the genus *Paraliobacillus*. Preliminary experiments have shown that *P. zengyii* exhibits significant antiviral activity [4], demonstrating its potential as a candidate antiviral probiotic. However, to ensure its safety as a novel probiotic strain, rigorous safety assessments must be conducted.

The current probiotic safety evaluation system includes four mainly stages: genomic bioinformatics analysis, in vitro experiments, evaluation in animal models, and human trials [5,6]. First, genomic bioinformatics analysis based on bacterial genome annotation can be performed to comprehensively analyze whether a strain carries potential pathogenic genes, antibiotic resistance genes, or virulence factor-related genetic elements [7]. In vitro experiments are used mainly to assess a strain’s biological characteristics, such as tolerance to the gastrointestinal environment, antibiotic sensitivity, hemolytic activity, and gelatinase activity [8]. *Lactobacillus paracasei* EG005, identified through genomic analysis and functional evaluations, exhibits enhanced antioxidant activity and represents a promising probiotic candidate [9]. Second, in animal model evaluation systems, rodents are widely used for safety assessment because of their physiological similarity to humans and relatively sensitive metabolic systems. Rodents have become the gold standard for evaluating the safety of drugs and probiotics [10,11,12]. The safety assessment of mice showed that *Bacillus subtilis* PBC01 caused no deaths or adverse effects on blood cell composition, antioxidant capacity, or reproductive health, demonstrating its excellent probiotic properties and safety [13]. Acute oral toxicity experiments in mice can be used to assess the acute toxicity response of probiotics to the host and provide a theoretical basis for further clinical trials. Although a widely validated probiotic safety evaluation system has not yet been established, animal oral toxicity experiments remain a common method to determine probiotic safety and constitute a core component of safety certification before probiotic products enter the market [14].

In this study, we revealed that *P. zengyii* possesses a diverse set of genes involved in carbohydrate and amino acid metabolisms. And we found that the strain exhibited a certain degree of tolerance to bile salts and acidic environments and no detectable gelatinase or hemolytic activity. Furthermore, our acute oral toxicity tests in mice revealed that *P. zengyii* does not induce any adverse effects. The integration of genomic analysis, in vitro experiments, and acute oral toxicity tests in mice provided a preliminary safety evaluation of *P. zengyii*, providing a theoretical basis for the development of novel functional probiotic strains.

## 2. Results

### 2.1. Genome Characteristics and Homology Analysis of P. zengyii

**Figure 1:**
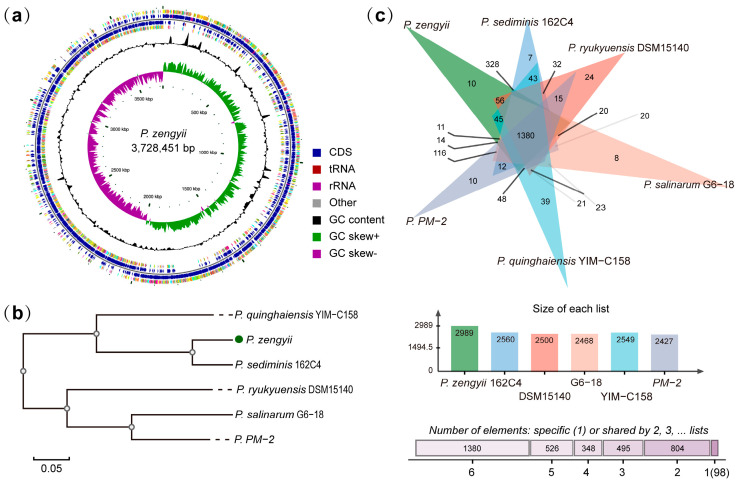
Genomic characteristics and homology analysis of *P. zengyii*. (**a**) Circular genome maps of *P. zengyii*. Starting from the outside, the first and fourth circles show CDSs on the positive and negative strands, respectively; the second and third circles show CDSs, tRNAs, and rRNAs on the positive and negative strands, respectively; the fifth circle shows the GC content; the sixth circle shows the GC-skew value; and the innermost circle shows the marker of genome size. (**b**) Phylogenetic tree based on the genomic protein sequences of type strains of *Paraliobacillus*. The numbers within brackets indicate the NCBI GenBank accession numbers for the genome sequences. (**c**) Classic Venn diagrams showing the selected strains.

Figure 1a shows that the genome size of *P. zengyii* is 3,728,451 bp, with a total GC content of 35.2%. Its genome contains 3575 protein-coding genes, 28 rRNA genes, and 64 tRNA genes. Multiple strains of the genus *Paraliobacillus* have been isolated from different sources. To explore the evolutionary relationships between *P. zengyii* and related species, six model strains were selected for the construction of a phylogenetic tree based on orthologous proteins. The results indicated that *P. zengyii* is most closely related to *P. sediminis* 162C4 (Figure 1b). Through OrthoVenn analysis, this study identified 1380 orthologous proteins shared by these strains. Further analysis revealed that *P. quinghaiensis* YIM-C158 has the greatest number of unique protein sequences (39), while *P. zengyii* and *P. PM-2* share the same number of unique protein sequences (10). Additionally, the genome of *P. zengyii* formed a greater number of clusters (2989) (Figure 1c).

### 2.2. Gene Annotation for P. zengyii

To further investigate gene functions in *P. zengyii,* functional annotation of the predicted coding genes was performed using the TCDB, CAZyme, antiSMASH, VFDB, KEGG, and GO databases. Table 1 shows that 738 *P. zengyii* genes were annotated in the TCDB, 113 in the CAZyme database, 5 in the antiSMASH database, 413 in the VFDB database, 2618 in the KEGG, and 458 in the GO database.

#### 2.2.1. TCDB Annotation

**Figure 2:**
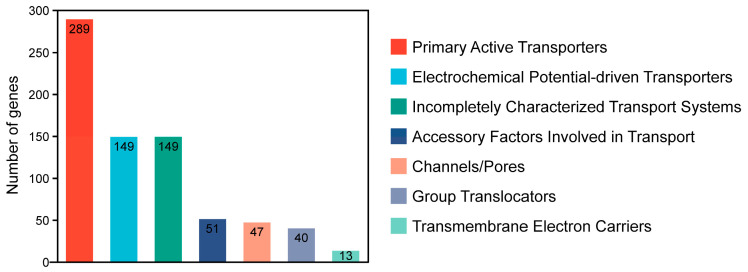
TCDB annotation of *P. zengyii* genes.

Figure 2 shows that the genome of *P. zengyii* was annotated in the TCDB, which identified a total of 738 transporter-related genes. These proteins are categorized into seven different classes on the basis of their transport mechanisms. Among them, primary active transporters were the most abundant, with 289 identified, followed by electrochemical potential-driven transporters and incompletely characterized transport systems, both with 149 annotations. The remaining categories included accessory factors involved in transport (51 annotated), channels/pores (47 annotated), group translocators (40 annotated), and transmembrane electron carriers, which were the least represented, with only 13 annotations. These results suggested that *P. zengyii* facilitates the exchange of chemical substances and signals across the biological membrane via multiple transport proteins.

#### 2.2.2. CAZyme Database Annotation

**Figure 3:**
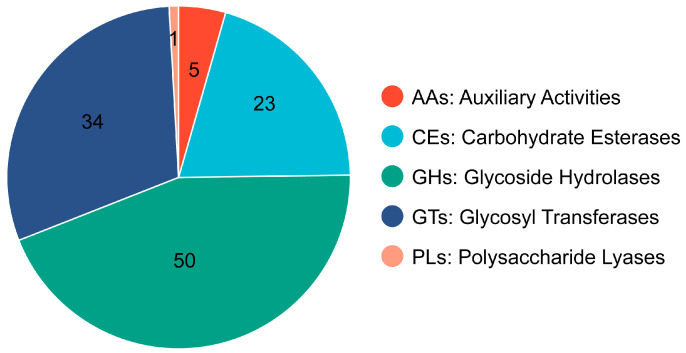
Distribution of CAZymes in *P. zengyii*.

Figure 3 shows the results of the alignment analysis of the *P. zengyii* genome data with the CAZyme database. A total of 113 genes in the *P. zengyii* genome encode protein domains belonging to the CAZyme family. Among these, glycoside hydrolases (GHs) are the most abundant, followed by glycosyl transferases (GTs), carbohydrate esterases (CEs), auxiliary activities (AAs), and polysaccharide lyases (PLs). These results suggest that the *P. zengyii* genome has the capacity to encode a variety of carbohydrate-active enzymes, which may play important roles in the degradation and synthesis of various carbohydrates.

#### 2.2.3. antiSMASH Database Annotation

The antiSMASH database analysis of the *P. zengyii* genome revealed that this strain contains a total of five metabolic gene clusters. The major types of gene clusters identified include clusters for terpenes, ectoine, type III polyketide synthases (T3PKSs), and lasso peptides (Table 2). Further BLAST analysis (https://blast.ncbi.nlm.nih.gov/Blast.cgi, accessed on 25 February 2025) was performed by comparing the five gene clusters of *P. zengyii* with known biosynthesis-related gene cluster sequences. The ectoine gene cluster shares 75% similarity with known biosynthesis-related gene clusters, whereas the lasso peptide gene cluster shares 50% similarity with the paeninodin biosynthesis-related gene cluster. These results suggest that the *P. zengyii* genome may harbor novel biosynthesis-related gene clusters for active compounds.

#### 2.2.4. VFDB Annotation

On the basis of the alignment results from the VFDB, the virulence factors in the *P. zengyii* genome were predicted in this study, and the predictions were largely consistent with previous results. However, the genes encoding flagellum-associated virulence factors predicted in this study presented less than 50% sequence identity. Virulence factors with ≥70% sequence identity were further analyzed, and a total of five virulence-related genes were annotated, including genes encoding the ClpC protein, the translation elongation factor EF-Tu, the molecular chaperone GroEL, the serine protease ClpP, and the capsule protein (Table 3). Among these proteins, ClpC is a protein belonging to the ATPase family that can form a complex with ClpP protease (ClpCP), and these proteins together constitute the bacterial protein degradation system. This system recognizes, unfolds, and delivers misfolded or damaged proteins to maintain intracellular protein homeostasis. ClpC plays an important role in bacterial responses to oxidative stress, nutrient deprivation, and other environmental pressures. GroEL is a molecular chaperone that forms a complex with GroES to facilitate the proper folding of newly synthesized or stress-affected proteins. EF-Tu is exposed on the bacterial surface and can bind to host extracellular matrix proteins such as fibronectin and laminin, promoting adhesion and invasion. The capsule helps bacteria resist phagocytosis by the host immune system, preventing recognition and clearance by the host immune system. In summary, these factors assist bacteria in surviving within the host.

#### 2.2.5. KEGG Database Annotation

**Figure 4:**
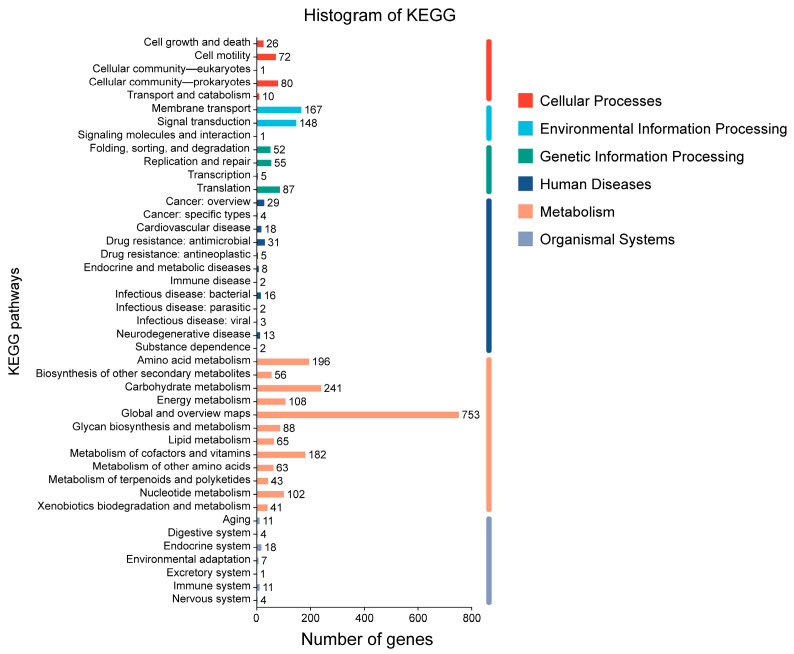
KEGG functional annotation of *P. zengyii* genes.

Through KEGG pathway annotation of the *P. zengyii* genome, a total of 2618 metabolic pathway genes were annotated, which is an increase of 674 genes compared with previous annotations. Figure 4 showed that the functionally annotated genes were categorized into six major groups: cellular processes, environmental information processing, genetic information processing, human diseases, metabolism, and organismal systems. Among these groups, metabolism-related genes accounted for the largest number, with 1938 genes annotated, whereas there were fewer genes related to organismal systems, with only 56 genes identified. Among the metabolic pathways, three major metabolic categories stood out: global overview (753 genes), carbohydrate metabolism (241 genes), and amino acid metabolism (196 genes). Compared with annotations in previous databases, there were significant differences in the number of genes involved in metabolic pathways in *P. zengyii*. Additionally, among environmental information processing pathways, relatively high numbers of genes were annotated in membrane transport pathways and signal transduction pathways (167 and 148 genes, respectively). The membrane transport pathways included ABC transporters, phosphotransferase systems, and bacterial secretion systems, all of which play key roles in cellular substance transport, external signal transduction, and feedback mechanisms.

#### 2.2.6. GO Database Annotation

**Figure 5:**
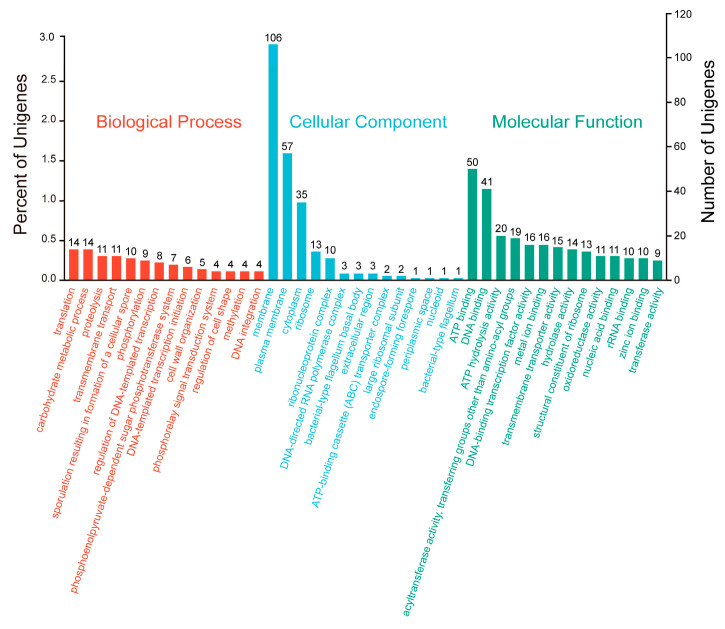
GO functional annotation of *P. zengyii* genes.

GO database annotation of the *P. zengyii* genes revealed the genes involved in three major GO categories: biological processes, cellular components, and molecular functions (Figure 5). A total of 458 genes were annotated, with significant differences in distribution across the categories. In the molecular function category, 331 genes were annotated, with ATP-binding genes being the most common (50), followed by DNA-binding genes (41). In the biological process category, 192 genes were annotated, primarily associated with translation and carbohydrate metabolism (14 genes each). Additionally, 11 genes each were annotated in proteolysis and transmembrane transport. In the cellular component category, 224 genes were annotated, with membrane-associated genes being the most abundant (106). The next largest group consisted of genes related to the cell membrane (35), further demonstrating the diversity of *P. zengyii* in terms of membrane structure and function. In summary, the GO annotation results for *P. zengyii* revealed the functional features of this strain in terms of multiple biological processes, including energy metabolism, protein synthesis, and membrane structure and function.

### 2.3. Antibiotic Sensitivity Analysis and Plasmid Detection Analysis of P. zengyii

**Figure 6:**
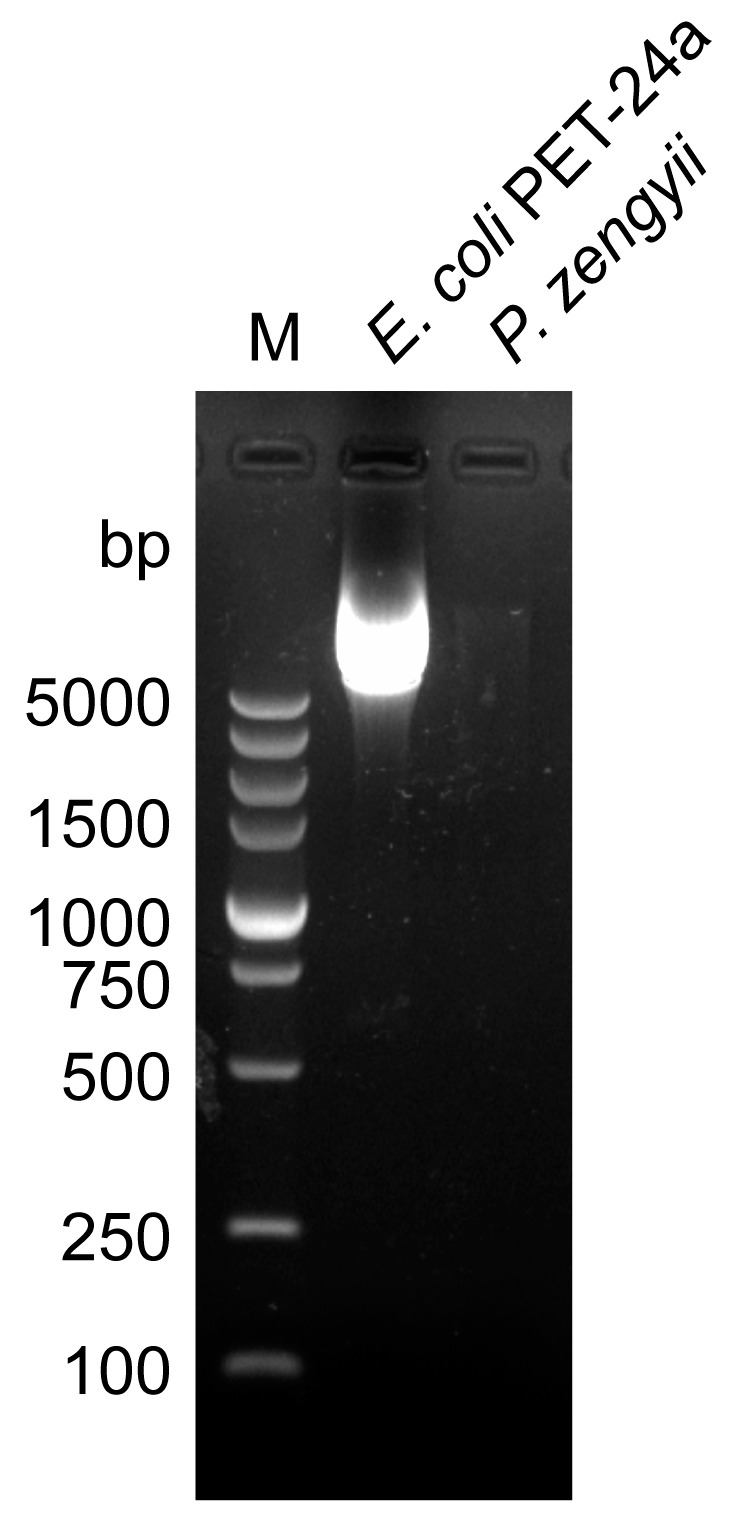
Agarose gel electrophoresis of plasmid DNA from *P. zengyii* and *E. coli* PET-24a. M: DL5000 DNA Marker.

The E test method was used to assess the antibiotic sensitivity of *P. zengyii* to 13 different antibiotics. The results revealed that *P. zengyii* was sensitive to tetracycline, clindamycin, erythromycin, tigecycline, amikacin, trimethoprim–sulfamethoxazole, azithromycin, chloramphenicol, ceftriaxone, ciprofloxacin, rifampicin, and daptomycin but resistant to polymyxin B (Table 4). Probiotics should not contain plasmids carrying antibiotic resistance genes, and whole-genome analysis revealed that *P. zengyii* does not carry plasmids. Further plasmid detection experiments confirmed that *P. zengyii* does not contain plasmids (Figure 6). These results indicate that the antibiotic resistance genes in *P. zengyii* are located on the chromosome and that there is no risk of horizontal gene transfer, further confirming the safety of *P. zengyii*.

### 2.4. Hemolytic Activity and Gelatinase Activity of P. zengyii

**Figure 7:**
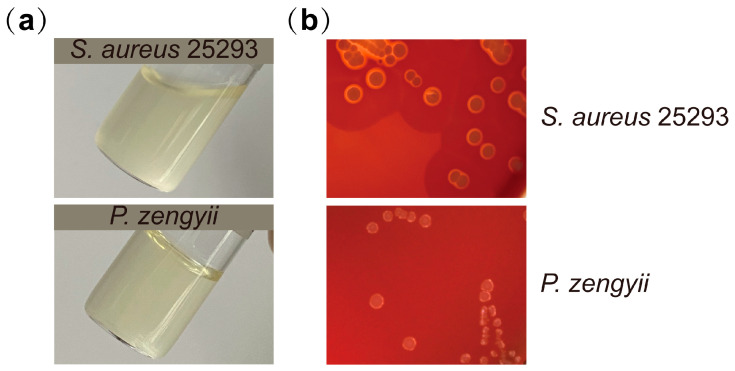
Hemolytic and gelatinase activities of *P. zengyii*. (**a**) Gelatinase activity of *P. zengyii*; *S. aureus* 25923 was used as a positive control. (**b**) Hemolytic capacity of *P. zengyii*; *S. aureus* 25923 was used as a positive control.

To further evaluate the safety of *P. zengyii,* its gelatinase and hemolytic activities were assessed. *P. zengyii* did not exhibit gelatinase activity, whereas *S. aureus* 25923, which was used as a positive control, tested positive for gelatinase, with gelatin being broken down to a liquid form (Figure 7a). In the hemolysis experiment, when *P. zengyii* and *S. aureus* 25923 were cultured on blood agar plates, a clear hemolytic zone was observed around *S. aureus* 25923, indicating its β-hemolytic activity. However, *P. zengyii* did not form a hemolytic zone, indicating the absence of hemolytic activity and suggesting that it does not induce adverse hemolytic reactions (Figure 7b).

### 2.5. Acid and Bile Salt Tolerances of P. zengyii

**Figure 8:**
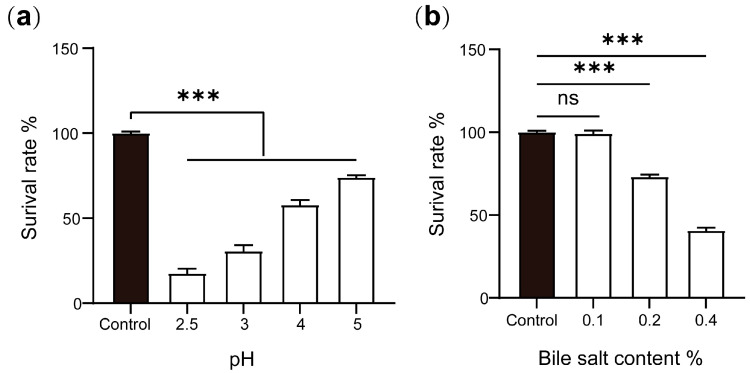
Acid and bile salt tolerances of *P. zengyii*. (**a**) Survival rate of *P. zengyii* under different pH conditions. (**b**) Survival rate of *P. zengyii* under different concentrations of bile salts. The data are presented as means ± SEMs. Significance was determined using one-way ANOVA. ns, no significant difference; *** *p* < 0.001.

The survival rates of *P. zengyii* at different pH values are shown in Figure 8a. At a pH of 2.5, the survival rate of *P. zengyii* was only 17.7%, whereas at pH 3, the survival rate increased to 30.9%. At pH values of 4 and 5, the survival rate exceeded 50%, with the survival rate reaching 74.3% at pH 5. These results indicate that *P. zengyii* can survive in low-pH environments, demonstrating its acid tolerance. Figure 8b shows the survival rate of *P. zengyii* under different bile salt concentrations. As the bile salt concentration increased, the survival rate of *P. zengyii* significantly decreased. At a bile salt concentration of 0.1%, *P. zengyii* presented a high survival rate. However, at bile salt concentrations of 0.2% and 0.4%, the survival rates decreased to 73.4% and 40.9%, respectively. These data suggest that *P. zengyii* has a certain level of bile salt tolerance and can survive in environments with moderate bile salt concentrations.

### 2.6. In Vivo Safety Evaluation of P. zengyii

**Figure 9:**
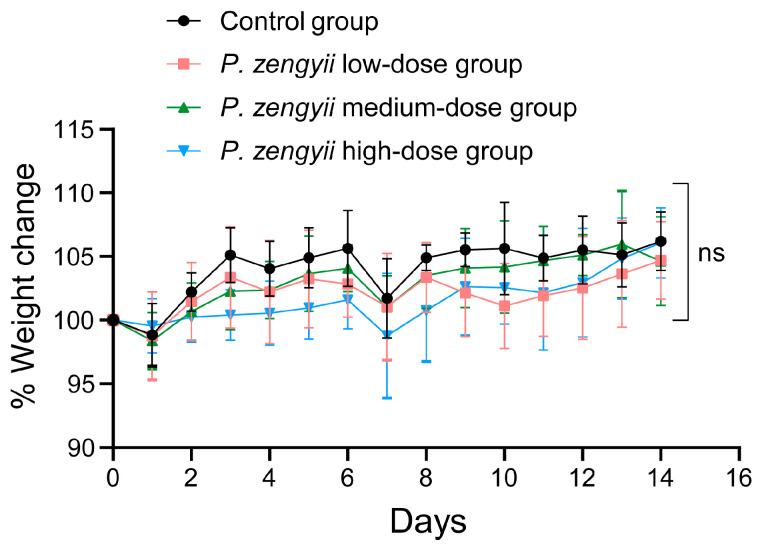
Changes in the body weights of the mice in each group. ns, no significant difference.

During the entire experiment, all the mice in the experimental groups were in good mental health, with no significant abnormalities in appearance, normal activity, and no issues with eating or breathing. Regardless of the dose of *P. zengyii* (low, medium, or high), the mice exhibited stable behavioral and physiological states. These findings indicate that at different doses, *P. zengyii* does not have a significant negative effect on the overall health of the mice or interfere with their basic physiological functions. The changes in the body weights of the mice over the 14-day experimental period are shown in Figure 9. Compared with those in the control group, there were no significant differences in body weight gain trends among the experimental groups (*p* > 0.05), that is, in the low-, medium-, or high-dose groups. This result suggested that within the tested dose range, *P. zengyii* did not affect the weight gain of the mice. Furthermore, these findings indicate that *P. zengyii* has no noticeable effect on the growth or development of the mice within this dose range, suggesting a high level of safety.

**Figure 10:**
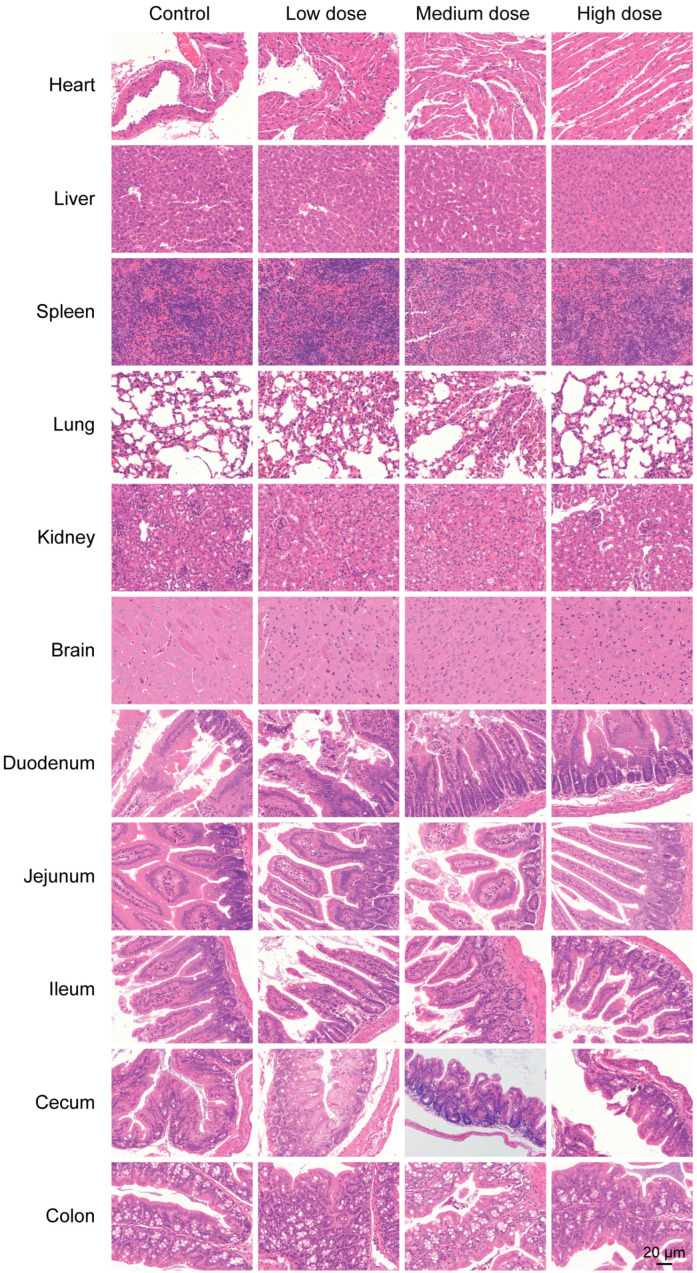
Pathological examination of major organs in the different *P. zengyii* dose groups via H&E staining. Scale bars, 20 μm.

After the experiment, the mice were euthanized, and their vital organs, including the heart, liver, spleen, lungs, and kidneys were examined. No significant pathological changes or abnormalities were observed. The organs were weighed, and organ indices were calculated (Table 5). Statistical analysis revealed that there were no significant differences in the organ indices between the experimental groups and the control group (*p* > 0.05). These findings indicate that *P. zengyii* intervention did not lead to changes in the weight of vital organs or potential toxicity in the mice, further supporting its safety at certain doses. To further evaluate whether *P. zengyii* causes potential damage to the major organs of mice, histopathological analysis with H&E staining was performed on the heart, liver, spleen, lung, kidney, brain, duodenum, jejunum, ileum, cecum, and colon. No significant histological changes, such as congestion, degeneration, necrosis, hyperplasia, or inflammation, were observed in the major organs of the three different *P. zengyii*-treated dose groups relative to those in the control group (Figure 10). These results suggest that *P. zengyii* did not cause significant pathological changes in the major organs of the mice, further confirming its safety in terms of organ health.

## 3. Discussion

On the basis of a phylogenetic tree constructed for orthologous proteins, this study revealed that *P. zengyii* is closely related to *P. sediminis* 162C4, which is consistent with previous phylogenetic tree analyses based on 16S rRNA [15]. *P. sediminis* 162C4 was isolated from marine sediments in the East China Sea [16], whereas *P. zengyii* was isolated from the Tibetan Plateau. Despite the large geographical distance between the two isolation sites, both are high-salinity environments. This suggests a potential link between the microbial communities of the Tibetan Plateau and the ocean.

Bacterial hemolysins are toxins produced by bacteria that directly lyse various cell types and disrupt vascular permeability, leading to bacterial proliferation within cells, the inhibition of lymphocyte proliferation, and the suppression of lymphokine and immunoglobulin production [17]. Hemolysis can cause host immune system abnormalities, such as anemia, and one of the basic requirements of probiotics is that they should not exhibit hemolytic activity [18,19]. In this study, no significant hemolytic zone was observed around the *P. zengyii* colonies, whereas a clear hemolytic zone (β-hemolysis) was observed around the positive control *S. aureus* 25923 colonies, indicating that *P. zengyii* does not cause hemolytic reactions. Additionally, some pathogenic bacteria secrete gelatinase to degrade the basement membrane of host tissues, promoting the spread of infection and increasing the risk of infecting other tissues [20,21]. This study demonstrated, through gelatin biochemical tube tests, that *P. zengyii* does not exhibit gelatinase activity, further confirming its safety.

In the whole-genome analysis, using the updated VFDB, 413 virulence factors were identified in *P. zengyii*, with over 70% consistency in the identification of five virulence factors. Notably, the analysis results are consistent with those of previous studies, and many of these virulence factors are related to gastrointestinal tolerance, such as genes encoding heat shock proteins and molecular chaperones (e.g., ClpC, GroEL, and ClpP), which help *P. zengyii* survive in the intestinal environment [22,23,24]. In acid and bile salt tolerance experiments, *P. zengyii* was able to grow under acidic conditions and in the presence of bile salts, indicating that the strain can survive in harsh environments, such as the gastrointestinal tract.

Antibiotic resistance is an important factor in the evaluation of the safety of probiotics. Antibiotic susceptibility tests revealed that *P. zengyii* is resistant to polymyxin. As a Gram-positive bacterium in the *Bacillus* family, *P. zengyii* lacks an outer membrane and lipopolysaccharide (LPS) in its cell wall, preventing the action of polymyxins [25,26], which is why most Gram-positive bacteria are resistant to polymyxins [27]. Plasmid-mediated polymyxin resistance is an acquired form of resistance that poses a serious global antibiotic resistance threat [28,29]. This study further confirmed, through plasmid detection experiments, that *P. zengyii* does not contain plasmids, suggesting that its antibiotic resistance is not plasmid mediated, thus posing no risk of the transfer of resistance genes.

Metabolic pathway prediction is another crucial aspect of probiotic research. According to the KEGG database, *P. zengyii* contains abundant genes related to carbohydrate and amino acid metabolisms, which are typical characteristics of probiotics [30]. In particular, the GH family is abundant in the *P. zengyii* genome, indicating its significant role in the hydrolysis of glycosidic bonds in carbohydrates. Furthermore, the genome analysis also contains predicted genes encoding enzymes such as GTs and CEs, which is consistent with the results for other candidate probiotics [30,31]. Importantly, carbohydrates can be metabolized to SCFAs, which serve as an additional energy source and play crucial roles in gut health and the gut–brain axis [32,33]. Through antiSMASH database analysis, *P. zengyii* was also found to produce ectoine, which can serve as a protectant and stabilizer for proteins and other biomolecules, helping the cells resist various adverse environmental factors, such as salinity, heat, dryness, freezing, thawing, and ionizing radiation [34]. Additionally, *P. zengyii* can metabolize and produce type III polyketide synthases, which can generate compounds with specific antibacterial activities; these compounds act as natural preservatives [35]. Furthermore, *P. zengyii* can metabolize and produce lasso peptides, which have significant antibacterial activity against Gram-positive bacteria such as methicillin-resistant *Staphylococcus aureus* (MRSA) [36]. Some lasso peptides also act as selective antagonists for specific receptors, e.g., endothelin β-receptor antagonists such as RES-701-1, and others exhibit antibacterial, antiviral, and antitumor activities [37,38]. Owing to their high stability and modifiability, lasso peptides can also serve as molecular scaffolds for the development of novel drugs [39]. Although the *P. zengyii* gene clusters share certain similarities with known gene clusters, the differences in similarity suggest that this strain may have unique metabolic potential and the ability to generate new natural products. In future research, further exploration of the functions of these gene clusters and their potential biological activities will be necessary, offering possibilities for discovering new natural products. These results indicate that *P. zengyii* has tremendous potential for applications in fields such as food, medicine, and in industrial settings.

To further verify the safety of *P. zengyii*, an in vivo safety evaluation was conducted in a mouse model. Two weeks after the oral administration of *P. zengyii*, no disease, death, or infection symptoms were observed in the mice at any dose. Additionally, histological examination with H&E staining did not reveal any pathological changes, such as inflammatory infiltration, tissue sclerosis, or lymphoid hyperplasia. In animal toxicity assessment experiments, body weight and organ indices are important indicators of adverse effects, providing a visual reflection of the impact of substances on animals [40]. Importantly, the analysis in this study revealed that *P. zengyii* did not affect the body weights or organ indices of the mice. However, while the acute toxicity results are promising, sub-chronic and chronic studies are critical to assess long-term safety, particularly for strains intended for clinical use. These studies will also provide insights into the optimal dosing regimen and population-specific considerations. Further evaluations of the strain’s safety are necessary moving forward.

## 4. Materials and Methods

### 4.1. Bacterial Strains and Culture Conditions

*P. zengyii* was isolated and stored in our laboratory [15]. The bacterial strain was routinely grown in BHI medium (Oxoid, Basingstone, UK) supplemented with 3% (*w/v*) NaCl (Solaibao, Beijing, China) at an optimal temperature of 28 °C. The passaged *P. zengyii* was inoculated for 12 h for activation. The activated culture was then transferred at a 1:100 dilution into fresh BHI media containing 3% (*w*/*v*) NaCl and further cultured with shaking for 6 to 8 h until the logarithmic growth phase was reached (OD_600_ = 0.6). The culture was subsequently centrifuged at 8000 rpm for 10 min to collect the cell pellet. The pellet was washed three times with PBS to remove residual culture medium, with the cells being resuspended in PBS after each wash. Following the final wash, the bacterial pellet was resuspended in PBS and adjusted to the desired experimental concentration for subsequent use. *Escherichia coli* containing the PET-24a plasmid (*E. coli* PET-24a) and *Staphylococcus aureus* (*S. aureus*) ATCC 25923 were stored in our laboratory. *E. coli* PET-24a and *S. aureus* 25923 were cultured in BHI medium at 37 °C. All three strains were grown under aerobic conditions.

### 4.2. Genomic Features and Phylogenetic Analysis

The genome sequence of *P. zengyii* was obtained from the NCBI database, with the species ID 2213194 and accession number GCF_003268595.1. In this study, Prodigal (version 2.6.3) was used to predict the coding sequences (CDSs) within the genome; tRNAscan-SE (Version 2.0) was used for transfer RNA (tRNA) prediction; Barrnap (https://github.com/tseemann/barrnap, accessed on 25 February 2025) was employed for ribosomal RNA (rRNA) prediction; and the Proksee online tool (https://proksee.ca/, accessed on 25 February 2025) was used to generate a circular map of the *P. zengyii* genome. A phylogenetic tree and whole-genome homologous gene comparison analysis were conducted using OrthoVenn3 (https://OrthoVenn3.bioinfotoolkits.net/home, accessed on 25 February 2025) across six closely related model strains within the genus. All genome sequences were retrieved from the NCBI database (https://www.ncbi.nlm.nih.gov/genome/, accessed on 20 January 2025), including those for *P. sediminis* 162C4 (GCF_003426055.1), *P. ryukyuensis* DSM15140 (GCF_003315295.1), *P. salinarum* G6-18 (GCF_014083865.1), *P. quinghaiensis* YIM-C158 (GCF_003426025.1), and *P. PM-2* (GCF_001368815.1).

### 4.3. Genome Functional Annotation

Preliminary functional annotation of the *P. zengyii* genome was performed by Wang et al. [15]. To further evaluate the functional stability and safety of *P. zengyii* as a potential probiotic, its genome was systematically analyzed in this study. By integrating multiple specialized databases, we explored the characteristics of its transport proteins, carbohydrate-active enzymes, secondary metabolite biosynthesis gene clusters, and metabolic pathways. Below are the primary databases and analytical methods used in this study. The Transporter Classification Database (TCDB) (http://www.tcdb.org/, accessed on 25 February 2025) was used to identify transport-related proteins in the genome; the Carbohydrate-Active Enzymes Database (CAZy) (http://www.cazy.org/, accessed on 25 February 2025) was used to predict carbohydrate-active enzymes that play a key role in bacterial metabolism; the Antibiotics & Secondary Metabolite Analysis Shell (antiSMASH) database (http://antismash.secondarymetabolites.org/, accessed on 25 February 2025) was utilized to predict secondary metabolite biosynthesis gene clusters in the genome; and the Gene Ontology (GO) database (http://www.geneontology.org/, accessed on 25 February 2025) was used to systematically describe and define the functions of genes and proteins, revealing gene functional characteristics. Furthermore, metabolic pathway analysis was performed using the latest version of the Kyoto Encyclopedia of Genes and Genomes (KEGG) (Version 2025.01). In addition, the Virulence Factor Database (VFDB) (Version 2024.03) was used to analyze potential virulence genes and assess the safety of *P. zengyii* as a probiotic.

### 4.4. Antibiotic Sensitivity Testing

In accordance with the guidelines of the European Food Safety Authority (EFSA), antibiotic susceptibility testing of *P. zengyii* was performed in this study using the E test (Haibo Biology, Qingdao, China) to determine its sensitivity to 13 antibiotics: tetracycline, clindamycin, polymyxin, erythromycin, tigecycline, amikacin, cotrimoxazole, azithromycin, chloramphenicol, ceftriaxone, ciprofloxacin, rifampicin, and quinupristin–dalfopristin. The *P. zengyii* bacterial suspension was evenly spread on the surface of BHI + 3% NaCl solid agar medium, and E test strips were placed on the surface of the agar. The culture was incubated at 28 °C for 48 h, during which the inhibitory effects of each antibiotic on *P. zengyii* were observed. The minimal inhibitory concentration (MIC) was determined to assess bacterial resistance. The MIC is the lowest antibiotic concentration that inhibits bacterial growth. Antibiotic sensitivity evaluation was performed in accordance with the standards for *Bacillus* species (excluding *Bacillus anthracis*) set by the Clinical and Laboratory Standards Institute (CLSI).

### 4.5. Plasmid Extraction and Detection

In this study, plasmid DNA was extracted from *P. zengyii* using a Gram-positive bacterial plasmid DNA extraction kit (Solaibao, China) following the manufacturer’s instructions. A plasmid mini-preparation kit (Solaibao, China) was used to extract plasmid DNA from *E. coli* PET-24a, which served as a positive control. The extracted plasmid samples were analyzed using electrophoresis on a 1% (*w*/*v*) agarose gel. Electrophoresis was performed in 1 × TAE buffer (Solaibao, China) for 20 min at a voltage of 100 V. After electrophoresis, the gel was stained with an ethidium bromide-based nucleic acid dye. The gel was then observed and photographed using a UV transilluminator (Benchtop UV Transilluminators, Waltham, MA, USA) to detect the presence of the plasmid.

### 4.6. Hemolysis Test

The stably passaged strain was streaked onto 5% (*w*/*v*) sheep blood agar plates (Haibo Biology, China) and incubated at an appropriate growth temperature for 24 to 48 h The hemolytic activity of the strain was determined by observing the hemolytic zone around the area of bacterial growth after 24–48 h of bacterial culture. The type of hemolysis was determined as follows: a greenish zone around the colony indicated α-hemolysis; a clear, colorless zone indicated β-hemolysis; and no hemolytic zone around the colony indicated γ-hemolysis, indicating that the strain lacked hemolytic activity. *S. aureus* 25923 was used as the positive control for the hemolysis test.

### 4.7. Gelatinase Activity Test

The gelatinase activity of *P. zengyii* was determined via the use of gelatin biochemical tubes (Haibo Biology, China). First, the biochemical tubes were preheated in a 37 °C incubator for 10 min to ensure that the medium in the tubes remained in a liquid state. After the biochemical tubes were opened under sterile conditions, 50 μL of bacterial suspension (2 × 10^7^ CFU) was added, and the tubes were incubated at 37 °C in a bacterial incubator. After 48 h, the biochemical tubes were placed in a 4 °C environment for 10 min to cool. The physical state of the medium was then observed by tilting the tube. If the medium in the tube remained solid, a negative result for gelatinase activity was indicated. If the medium remained liquid, a positive result was indicated. *S. aureus* 25923 was used as the positive control.

### 4.8. Acid Resistance Test

BHI medium containing 3% (*w*/*v*) NaCl was treated with HCl solution to achieve pH values of 2.5, 3.0, 4.0, and 5.0, and the medium was then filtered. In accordance with the methods of Liu et al. [41], the activated *P. zengyii* suspension was inoculated into liquid media with different pH values and incubated at 37 °C for 24 h. The bacterial suspension was then transferred to a 96-well plate, and the optical density (OD) value at a wavelength of 490 nm was measured using a multifunctional microplate reader (Biotek Instruments Inc., Winooski, VT, USA). *P. zengyii* cultured in medium without pH adjustment was used as the control. The survival rate = (OD value of the experimental group/OD value of the control group) × 100%

### 4.9. Bile Salt Tolerance Test

Bovine bile salt (Haibo Biology, China) was added to BHI liquid medium containing 3% (*w*/*v*) NaCl to yield bile salt concentrations of 0.1%, 0.2%, and 0.4%. Medium without bovine bile salt served as the control group. All media were filtered, sterilized, and set aside for use. The activated *P. zengyii* suspension was inoculated into liquid media with different bile salt concentrations and incubated at 37 °C for 24 h. The growth of *P. zengyii* in media with different bile salt concentrations was assessed by measuring the OD value. The measurement method was the same as that described in Section 4.8.

### 4.10. Animal Experiments

Five- to six-week-old female C57BL/6J mice were purchased from Vital River Laboratory Animal Technology Co., Ltd. (Beijing, China) and were maintained at a constant temperature in a humidity barrier system. During the experiment, all the mice had free access to food and water, and both the food and water met the standard requirements for laboratory animals. After the mice had acclimatized to the environment for one week, they were randomly divided into four groups: the control group (control, gavaged with PBS), high-dose *P. zengyii* group (gavaged with *P. zengyii* at 1 × 10^10^ CFU/mouse), medium-dose *P. zengyii* group (gavaged with *P. zengyii* at 1 × 10^9^ CFU/mouse), and low-dose *P. zengyii* group (gavaged with *P. zengyii* at 1 × 10^8^ CFU/mouse), with 6 mice in each group. The gavage volume was 200 μL per mouse per day. Gavage treatment was conducted every day for 14 consecutive days. During the experiment, the clinical symptoms of the mice, including mortality, skin and fur conditions, mucosal color, respiratory rate, body movement coordination, and behavior patterns, were observed and recorded daily. The body weight was recorded daily from the beginning of gavage (day 0). After the intervention, the major organs (heart, liver, spleen, lungs, and kidneys) were collected and examined macroscopically, and the weight of each organ was recorded to calculate the organ index. Additionally, tissue samples from various organs (heart, liver, spleen, lung, kidney, brain, duodenum, jejunum, ileum, cecum, and colon) were collected, fixed in 4% paraformaldehyde (LabLead, Beijing, China), and prepared into tissue sections for hematoxylin and eosin (H&E) staining and simultaneous pathological evaluation. H&E staining was performed according to previous reports [42]. The organ index = organ weight/body weight.

### 4.11. Statistical Analysis

All the data are presented as the means ± standard deviations (means ± SDs) and were analyzed using GraphPad Prism 8.0 statistical software. For comparisons between multiple groups, one-way ANOVA was used, and comparisons between two groups were performed using Student’s *t* test. Here, * *p* < 0.05; ** *p* < 0.01; *** *p* < 0.001; and ns indicates no significant difference.

## 5. Conclusions

This study comprehensively evaluated the genomic characteristics and safety of *P. zengyii* as a potential probiotic through genomic bioinformatics analysis, in vitro experiments, and in vivo mouse studies. Genomic analysis revealed that *P. zengyii* possesses a rich set of genes related to carbohydrate and amino acid metabolisms, which is characteristic of probiotics. Additionally, *P. zengyii* is capable of producing natural compounds with antibacterial activity, antiviral activity, and other bioactivities, such as ectoine, type III polyketide synthases, and lasso peptides. The study also revealed that *P. zengyii* carries genes associated with gastrointestinal tolerance, including genes encoding the ClpC, GroEL, and ClpP proteins. Its intrinsic resistance is demonstrated by its tolerance to polymyxin, and in vitro experiments confirmed that it does not harbor plasmids, posing no risk of antibiotic resistance gene transfer. *P. zengyii* is highly tolerant to bile salts and acidic environments and does not exhibit gelatinase or hemolytic activity. Acute gastric gavage toxicity tests in mice revealed that *P. zengyii* did not cause any changes in body weight, organ indices, or tissue morphological damage at three different doses. In conclusion, this study demonstrated that *P. zengyii* has characteristics of potential probiotics in terms of both safety and functionality.

## Figures and Tables

**Table 1:** Statistical analysis of *P. zengyii* gene annotations.

Database	TCDB	CAZyme	antiSMASH	VFDB	KEGG	GO
Number of genes	738	113	5	413	2618	458

**Table 2:** Analysis of secondary metabolite gene clusters of *P. zengyii*.

Cluster ID	Type	Start-End	Similar Cluster	Similarity (%)
Cluster 1	terpene	294,564–314,354	-	-
Cluster 2	ectoine	315,647–326,034	ectoine	75
Cluster 3	terpene	1,809,113–1,829,932	-	-
Cluster 4	T3PKS	1,961,117–2,002,290	-	-
Cluster 5	lasso peptide	3,062,131–3,084,506	paeninodin	50

**Table 3:** VFDB annotation of *P. zengyii* genes.

Gene ID	VFDB ID	VF	VF Category	Related Gene	Identity (%)
Gene 0139	VFG000079 (gb|NP_463763)	ClpC (VF0072)	Stress survival	ClpC	75.9
Gene 0167	VFG046465 (gb|WP_003028672)	EF-Tu (VF0460)	Adherence	TufA	75.6
Gene 0501	VFG012095 (gb|WP_003435012)	GroEL (VF0594)	Adherence	GroEL	70.6
Gene 2841	VFG000077 (gb|NP_465991)	ClpP (VF0074)	Stress survival	ClpP	73.6
Gene 3151	VFG001301 (gb|WP_000459062)	Capsule (VF0003)	Immune modulation	Cap8E	71.8

**Table 4:** Sensitivity of *P. zengyii* to different antibiotics.

Antibiotic Class	Antibiotic	MIC (μg/mL)	Sensitivity ^1^
tetracyclines	tetracycline	0.0940	S
lincomycin	clindamycin	0.050	S
polypeptide antibiotics	polymyxin	6	R
macrolides	erythromycin	0.016	S
glycylcyclines	tigecycline	0.023	S
aminoglycosides	amikacin	16	S
sulfonamides	cotrimoxazole	0.019	S
macrolides	azithromycin	0.38	S
chloramphenicol	chloramphenicol	3	S
cephalosporins	ceftriaxone	0.016	S
quinolones	ciprofloxacin	0.064	S
rifamycin	rifampicin	0.016	S
streptogramins	quinupristin–dalfopristin	0.75	S

^1^ S, susceptible; R, resistant.

**Table 5:** Organ indices of the mice in each group.

Group	Heart (%)	Liver (%)	Spleen (%)	Lung (%)	Kidney (%)
Control group	0.52 ± 0.06	3.66 ± 0.35	0.25 ± 0.05	0.65 ± 0.08	0.9 ± 0.18
*P. zengyii* low-dose group	0.54 ± 0.05	3.64 ± 0.14	0.29 ± 0.04	0.62 ± 0.09	0.92 ± 0.06
*P. zengyii* medium-dose group	0.55 ± 0.04	3.53 ± 0.17	0.29 ± 0.1	0.65 ± 0.05	0.86 ± 0.09
*P. zengyii* high-dose group	0.57 ± 0.04	3.57 ± 0.1	0.24 ± 0.03	0.63 ± 0.15	0.87 ± 0.06

## Data Availability

The original contributions presented in the study are included in the article; further inquiries can be directed to the corresponding author.
